# Editorial: Cannabidiol Treatment in Neurotherapeutic Interventions

**DOI:** 10.3389/fphar.2021.752292

**Published:** 2021-10-18

**Authors:** Gustavo Gonzalez-Cuevas, Maria S Garcia-Gutierrez, Francisco Navarrete, Giordano de Guglielmo, Jorge Manzanares

**Affiliations:** ^1^ Department of Clinical Psychopharmacology, College of Pharmacy, Idaho State University, Meridian, ID, United States; ^2^ Instituto de Neurociencias Universidad Miguel Hernandez-CSIC, San Juan de Alicante, Spain; ^3^ University of California, San Diego, San Diego, CA, United States

**Keywords:** cannabidiol, neurotherapeutic, treatment, CBD-cannabidiol, neuropsychiatric disorders (NPD)

Cannabidiol, usually referred to as CBD, is the second most abundant active ingredient in cannabis, one of the oldest medicinal plants in the world (Zuardi, 2006). Although CBD was first extracted from cannabis in 1940 (Adams et al., 1940), its chemical structure was not fully characterized until 1963 (Mechoulam and Shvo, 1963). In terms of its pharmacokinetic profile, CBD is highly lipophilic, have poor oral bioavailability (as low as 6%), and is well tolerated by humans with no signs of toxicity or serious side effects and drug interactions (Millar et al., 2018; Huestis et al., 2019). Pharmacodynamically, CBD acts on over 65 molecular targets, including transient receptor potential vanilloid (TRPV) channels, serotonin (5-HT_1A_) receptors, and cannabinoid-related receptors such as G protein-coupled receptor 55 (GPR55). Interestingly, the actions of CBD on the two main endocannabinoid receptors are limited by its low affinity. At higher doses, it functions as a negative allosteric modulator for CB_1_ receptors and an inverse agonist for CB_2_ receptors. Indirectly, CBD also activates CB_1_ receptors by inhibiting fatty acid amide hydrolase (FAAH), the enzyme that degrades anandamide (AEA), the endogenous ligand for CB_1_ receptors (Britch et al., 2021). It is important to note that, unlike tetrahydrocannabinol (i.e., THC, the main psychoactive chemical in cannabis), CBD is non-addictive (Viudez-Martinez et al., 2019), which makes it an exceptional alternative to THC-derivative cannabinoid drugs.

Touted as a cure-all for many health conditions and disorders (e.g., anxiety, depression, schizophrenia, PTSD), CBD has become increasingly ubiquitous in the marketplace (Brown and Winterstein, 2019). Spurred by the increasing legality of the medical use of the Cannabis sativa plant, a number of medical benefits of CBD have been reported. In the U.S. specifically, CBD (Epidiolex^®^) is currently marketed for the treatment of Dravet and Lennox-Gastaut syndromes, pediatric epilepsies resistant to anticonvulsants, as well as for spasticity in multiple sclerosis (Sativex^®^, THC:CBD). Emerging evidence from basic and clinical research suggests a relevant role for CBD in treating a variety of neuropsychiatric disorders, including schizophrenia (Osborne et al., 2017), mood disorders (Pinto et al., 2020), PTSD (Bitencourt and Takahashi, 2018), and drug addiction (Gonzalez-Cuevas et al., 2018; Viudez-Martinez et al., 2018), among others. However, very little is still known regarding the precise neurobiological mechanisms, pharmacokinetics, drug interactions, and clinical consequences of CBD treatment in many of these psychiatric conditions.

Despite the current CBD “boom” in commercially available products, often at the edge of the law (Mead, 2017), it remains to be investigated if CBD is an effective medical treatment for a wide range of neuropsychiatric conditions. In this special issue “Cannabidiol Treatment in Neurotherapeutic Interventions,” we present a series of reviews and research papers written by leading authors in the field of neuropsychopharmacology, providing a deep overview and analysis of scientifically sound evidence that evaluates the use of CBD alone or associated with another drug as a new therapeutic tool for the treatment of mental disorders.

Regarding the role of CBD in epilepsy, Dubois et al. evaluate a volumetric absorptive microsampling (VAMS) method combined with LC-MS/MS (liquid chromatography coupled to tandem mass spectrometry) that allows quantification of CBD blood levels, offering valuable support for personalized therapy in refractory epilepsy, and Contin et al. report the first clinical pharmacokinetic study in patients with Dravet and Lennox-Gastaut syndrome. Furthermore, Raucci et al. demonstrate the role of the endocannabinoid system in epileptogenesis and alert about the need to conduct double-blinded placebo-controlled trials about CBD efficacy and safety. Exploring the role of CBD in Alzheimer’s disease, Coles et al. find supporting evidence of CBD treatment potential for ameliorating cognitive impairments associated with this disease. Related to the potential treatment of schizophrenia with CBD, Leweke et al. show that CBD improves neurocognitive functioning in schizophrenics and Loss et al. point out to the CBD’s beneficial potential for the neurodevelopmental disorders of schizophrenia as well as autism spectrum disorders. For CBD research on mood disorders, Gasparyan et al. report that the combination of CBD and sertraline attenuates PTSD-related behavioral disturbances in mice, while normalizing gene expression alterations. Finally, a number of articles review the potential treatment of CBD for neuropsychiatric interventions: Navarrete et al. summarize the key involvement of CBD in the therapeutic intervention for Substance Use Disorders, Batalla et al. provide an overview of the neuroimaging studies in which CBD modulate functional networks relevant for psychiatric disorders, Patricio et al. focus on the neurobiological mechanisms of the CBD actions in the treatment of Parkinson’s disease and l-dopa-induced dyskinesias, Scarante et al. explore the contribution of glial cells to CBD effects in neuropsychiatric disorders, and Martinez-Orgado et al. assess the neuroprotective effects of CBD against Hypoxic-Ischemic Brain Injury (HIBI) in preclinical studies.

Neuropsychiatric illness, currently accounting a third of adult disability worldwide (Lake and Turner, 2017), will become the next major global health challenge unless new neurotherapeutics can be proven to provide clinical benefit (Insel, 2012). These are exciting times for research on CBD since it has demonstrated a wide range of promising therapeutic applications in preclinical studies, including the treatment of neuropsychiatric disorders. The current findings make this drug an attractive candidate for future clinical use and warrant further investigations. Only time, coupled with methodologically rigorous clinical trials, shall reveal the extent CBD interventions contribute to winning the fight against the mental pandemic of the 21st century.
